# A Medical Opinion of the Electric Light

**Published:** 1883-02

**Authors:** 


A Medical Opinion of the Electric Light—Before the electric light becomes, as it must soon become, the common illuminating agent of the period, says the *Lancet*, a determined effort should be made to devise some mode of mitigating its peculiarly unpleasant intensity. The vibratile impulse of the electric force is obviously stronger than the delicate terminal elements of the optic nerve in the retina can bear without injury. We are wont to apply the adjectives "hard" and "soft" to light, and their significance makes them peculiarly appropriate. The electric light is too hard ; it needs to be softened. The waves of motion are too short, and the outstroke—so to say—joins the instroke at too acute an angle. This might doubtless be obviated by employing suitable material for globes and shades, but perhaps the best plan would be to break up and scatter the rays of light by reflection. If a small convex reflector were placed immediately below the light in the protecting globe, and one of larger dimensions above it, so as to secure a double reflection with ultimate divergence downward and outward, the effect would be to cause the "rays" of light to fall obliquely on all objects within the immediate area of illumination. This would, perhaps, obviate the need of colored glasses, which the promoters of the electric light seem to dislike. Certainly there is a considerable sacrifice of power in the use of the opaline globe—so much, indeed, that some of the districts lighted by electricity displayed through this medium do not present any obvious superiority over gas. We throw out the suggestion for what it is worth. Something must be done, for, as it is, the electric light is "trying to the eyes," which means that it is in danger of injuring them, and already, there is reason to believe, mischief has been wrought by its use. For true comfort there is nothing like the light given by the old-fashioned pure wax candle.—*The Electrician.*

